# Rates of prostate surgery and acute urinary retention for benign prostatic hyperplasia in men treated with dutasteride or finasteride

**DOI:** 10.1186/s12894-016-0170-6

**Published:** 2016-08-31

**Authors:** Josephina G. Kuiper, Irene D. Bezemer, Maurice T. Driessen, Averyan Vasylyev, Claus G. Roehrborn, Fernie J. A. Penning-van Beest, Ron M. C. Herings

**Affiliations:** 1 PHARMO Institute for Drug Outcomes Research, van Deventerlaan 30-40, 3528 AE Utrecht, Netherlands; 2 GlaxoSmithKline, 980 Great West Road, TW8 9 GS Brentford London, UK; 3 GlaxoSmithKline, 980 Great West Rd, Brentford London, UK; 4 Department of Urology, University of Texas Southwestern Medical Center, 5323 Harry Hines Bldv, TX 75390 Dallas, TX USA

**Keywords:** Benign prostatic hyperplasia, Prostate surgery, Acute urinary retention, 5-alpha reductase inhibitors, Alpha-blocker

## Abstract

**Background:**

Previous studies have suggested a greater benefit for various outcomes in men diagnosed with benign prostatic hyperplasia (BPH) who are treated with dutasteride than for men treated with finasteride. This study investigates whether the rates of BPH-related prostate surgery and acute urinary retention (AUR) differ between dutasteride and finasteride users in the Netherlands.

**Methods:**

From the PHARMO Database Network, men aged ≥50 years with a dispensing of dutasteride or finasteride with or without concomitant alpha-blocker treatment between March 1, 2003 and December 31, 2011 were selected. The incidence of BPH-related prostate surgery and AUR was determined during dutasteride or finasteride treatment and stratified by type of initial BPH-treatment (5-ARI monotherapy or combination with alpha-blocker) and prescriber (general practitioner (GP) or urologist). Comparison of the incidence of BPH-related prostate surgery and AUR between the treatment groups was done by Cox proportional hazard regression.

**Results:**

11,822 dutasteride users and 5,781 finasteride users were identified. Most users started treatment in combination with an alpha-blocker. Overall, dutasteride users had a lower risk of BPH-related prostate surgery was lower among dutasteride users than finasteride users (HR: 0.75; 95 % CI: 0.56–0.99). This lower risk among dutasteride users was also seen when stratifying by monotherapy or combination therapy (HR: 0.73; 95 % CI: 0.54–0.98 for monotherapy and HR: 0.85; 95 % CI: 0.74–0.97 for combination therapy). However, the association was only present among men treated by urologists. For AUR the rates were low and no statistical significant difference was observed between dutasteride and finasteride users.

**Conclusions:**

The risk of undergoing BPH-related prostate surgery was lower among men using dutasteride compared to men using finasteride. The association was observed for monotherapy as well as combination therapy, however, only among men who received their prescription from a urologist.

## Background

Benign prostatic hyperplasia (BPH) affects 32–52 % of men aged 51–60 years and 77–99 % of men ≥81 years of age [1, 2]. Lower urinary tract symptoms (LUTS), such as poor stream and hesitancy, are common in patients with BPH. Progression of BPH may result in acute urinary retention (AUR) and need for surgical procedures that (partially) remove the prostate [3]. In order to relief BPH related symptoms, alpha-adrenoreceptor antagonists (“alpha-blockers”) can be used. Previous studies suggests that the combination of alpha-blockers and 5-alpha reductase inhibitors (5-ARIs) can be beneficial in the treatment of BPH associated with LUTS [4]. The greatest efficacy for this combination treatment was shown in patients with a large prostate size, a prostate-specific antigen (PSA) value of > 1.5 ng/ml, and with moderate to severe symptoms based on the International Prostate Symptom Score (IPSS). They experienced significant symptom relief and a decreased risk of AUR and surgery compared to patients treated with monotherapy [5]. Dihydrotestosterone (DHT), a metabolite of testosterone is the main mediator of prostate growth. As a class, 5-ARIs aimed to reduce the size of the prostate by blocking the activity of 5-alpha reductase enzymes in converting testosterone to DHT [6, 7]. The currently available 5-ARI agents in the United States and European market (dutasteride and finasteride), have been shown to reduce prostate volume by 20–30 % [8]. Long-term use of 5-ARI agents results in symptomatic improvements and reduction of the risk for AUR and prostate surgery [6, 9, 10]. Dutasteride blocks the type 1 as well as type 2 5-alpha reductase isoenzymes whereas finasteride blocks only the type 2 isoenzyme. Suppression of serum DHT has been shown to be more than 90 % by dutasteride and 70 % by finasteride [7].

In retrospective studies, the efficacy of finasteride and dutasteride has been compared for AUR and BPH-related surgeries showing significantly lower rates of AUR and BPH-related surgeries among dutasteride treated patients as compared with finasteride treated patients [11]. In another study, patients treated with dutasteride were less likely to experience AUR and prostate surgery than patients treated with finasteride [12]. A previous study conducted in Italy showed that the incidence of BPH-related hospitalization was lower among patients treated with dutasteride compared to patients treated with finasteride [13].

The purpose of this study was to compare the rates of BPH-related prostate surgery and AUR between dutasteride and finasteride alone or in combination with an alpha-blocker in the Dutch setting.

## Methods

### Data source

Data were obtained from the PHARMO Database Network in the Netherlands. This population-based network of healthcare databases combines data from different healthcare settings. Data sources are linked on a patient level through validated algorithms. The longitudinal nature of the PHARMO Database Network system enables to follow-up more than 4 million (25 %) residents of a well-defined population in the Netherlands for an average of ten years. For this study the Hospitalisation Database, the Out-patient Pharmacy Database and the General Practitioner (GP) Database were used. The Hospitalisation Database comprises hospital admissions from the Dutch Hospital Data Foundation [14], i.e., admissions for more than 24 h and admissions for less than 24 h for which a bed is required. The records include information about discharge diagnoses, procedures, and hospital admission and discharge dates. Diagnoses are coded according to the International Classification of Diseases [15] and procedures are coded according to the Dutch Classification of Procedures [16]. The Out-patient Pharmacy Database comprises GP or specialist prescribed healthcare products dispensed by the out-patient pharmacy. The dispensing records include information about type of medicine, dispensing date, strength, dosage regimen, quantity, route of administration, and prescriber specialty. Drug dispensings are coded according to the World Health Organization (WHO) Anatomical Therapeutic Chemical (ATC) Classification System. The GP Database comprises data from electronic patient records registered by GPs. The records include information on diagnoses and symptoms, laboratory test results, referrals to specialists and healthcare product/drug prescriptions. Diagnoses and symptoms are coded according to the International Classification of Primary Care (ICPC) [17]. The prescription records include information on type of product, date, strength, dosage regimen, quantity and route of administration. Drug prescriptions are coded according to the WHO ATC Classification System (WHO Anatomical Therapeutic Chemical Classification System) [18].

### Study population

From the Out-patient Pharmacy Database, men aged 50 years or older who were treated with dutasteride or finasteride between March 1, 2003 and December 31, 2011 were selected for inclusion in this study. The date of the first dispensing was designated as cohort entry date. Men were classified as either receiving 5-ARI monotherapy or 5-ARI in combination with an alpha-blocker at cohort entry date. 5-ARI in combination with an alpha-blocker was defined as continuous use of an alpha-blocker at cohort entry date or starting an alpha-blocker within 7 days of cohort entry date. The alpha-blocker and 5-ARI could be used as separate preparations or as combination pill. From the selected population, we excluded 1) men using 1 mg finasteride tablets (as these were indicated for the treatment of alopecia), and 2) men who had a hospitalization for prostate surgery, urinary retention, prostate cancer or bladder cancer before cohort entry date. To be able to define comorbidities in recent history and the risk of BPH-related prostate surgery, users were required to have continuous enrollment in the PHARMO Database Network of at least 12 months before and 12 months after the index date. All men were followed from cohort entry until moving out of the PHARMO catchment region, death, or end of the study period (December 31, 2011), whichever occurred first.

### Type of treatment

Dispensings of dutasteride, finasteride and alpha-blocker at cohort entry date and during follow-up were converted into treatment episodes of uninterrupted use. For each drug dispensing the duration of use was calculated by dividing the number of units dispensed by the number of units to be used per day, as defined in the dispensing records. In case of an interruption between two dispensings, use was considered uninterrupted if the duration of this gap was less than half the period of the given dispensing, or seven days, whichever is greater, according to the method of Catalan [19]. Episodes of uninterrupted use were constructed on the level of 5-ARI (dutasteride and finasteride episodes) and on the level of total BPH treatment (dutasteride monotherapy, finasteride monotherapy, dutasteride combination therapy or finasteride combination therapy). The first episode of 5-ARI treatment ended at the dispensing date of the alternative 5-ARI. The first episode of BPH treatment ended when the dutasteride or finasteride episode ended, when the alpha-blocker episode ended (for men on combined therapy) or when an alpha-blocker was dispensed (for men on monotherapy). Adherence with 5-ARI treatment during the first episode of BPH treatment was reflected by the medication possession rate (MPR), defined as the total number of days of 5-ARI treatment, divided by the duration of episode.

### Study endpoint

The primary endpoints in the current study were the rate of BPH-related prostate surgery and AUR during treatment with dutasteride compared to finasteride. BPH-related prostate surgery was defined by surgical procedures on the prostate during hospitalization with a primary or secondary discharge diagnosis of BPH (either coded as benign neoplasm or hyperplasia) and no diagnosis of prostate cancer. AUR was defined as a hospital admission with a primary or secondary discharge diagnosis of urinary retention.

BPH-related prostate surgery and AUR were determined during the first episode of 5-ARI treatment. Sensitivity analyses were performed by adding a washout period of 6 months after discontinuation of 5-ARI treatment to the observation period, and by limiting the observation period to the first episode of BPH treatment, i.e. ending observation upon addition (for initial monotherapy) or discontinuation (for initial combination therapy) of alpha-blocker therapy.

### Data analysis

Incidence rates of BPH-related prostate surgery and AUR were calculated by dividing the number of men with the event under investigation by the person-time at risk of the event under investigation in the population. Rates were stratified by concomitant use of alpha-blocker (mono- or combination therapy). As the setting of care is an indicator of severity of disease and may as well be related to surgery rates, results were also stratified by initial prescriber (GP or urologist). Hazard ratios (HR) and confidence intervals (CI) for comparison between the treatment groups were calculated using Cox proportional hazards regression adjusting for confounders. Potential confounders were age, chronic disease score (CDS), prescriber, adherence with 5-ARI treatment (medication possession rate (MPR)), history of bladder/kidney stones, urological care, haematuria, PSA, duration of alpha-blocker use, hypertension, hypercholesterolemia, diabetes type I, diabetes type II, Parkinson’s disease, multiple sclerosis, number of hospital admissions, number of drug dispensings (of any drug), and number of GP visits. Potential covariates that were associated with the outcome at an alpha of 5 % (univariate *p* < 0.05) and also were associated with choice of 5-ARI (t-test *p* < 0.05 for continuous variables and chi-square *p* < 0.05 for categorical variables) were included in the multivariate model if they changed the association between treatment and outcome by at least 5 %. All HRs were adjusted for geographic location to account for potential missing outcomes in combination with local prescriber preference.

All data were analysed using SAS programs organized within SAS Enterprise Guide version 4.3 (SAS Institute Inc., Cary, NC, USA) and conducted under Windows using SAS version 9.2.

## Results

### Patient characteristics

A total of 11,822 men treated with dutasteride (8,675 men (73 %) on combination therapy) and 5,781 men treated with finasteride (3,517 men (61 %) on combination therapy) between March 1, 2003 and December 31, 2011 were included in the analysis (Table 1). Most men on combination therapy were already using an alpha-blocker when they started 5-ARI (68 % of the dutasteride users and 75 % of the finasteride users). The mean age among the different cohorts was approximately 70 years. The initial prescriber of the first dutasteride dispensing was a urologist (57 %), while the first finasteride dispensing was primarily issued by the GP (49 %). The proportion of men that had received prior urological care was higher among dutasteride users than among finasteride users. The duration of the first 5-ARI treatment episode was on average 17 months for men receiving monotherapy of either finasteride or dutasteride. For men on combination therapy, the duration of the first 5-ARI treatment episode was on average 20 months for men on finasteride combination therapy and 18 months for men on dutasteride combination therapy.

**Table 1:** General characteristics of men with BPH using finasteride or dutasteride

	Dutasteride		Finasteride	
	monotherapy	& alpha-blocker	monotherapy	& alpha-blocker
	*N* = 3,147	*N* = 8,675	*N* = 2,264	*N* = 3,517
	n (%)	n (%)	n (%)	n (%)
Age at cohort entry				
50–54 years	150 (5)	330 (4)	172 (8)	148 (4)
55–59 years	320 (10)	860 (10)	255 (11)	371 (11)
60–64 years	468 (15)	1,541 (18)	341 (15)	577 (16)
65–79 years	1,659 (53)	4,681 (54)	1,101 (49)	1,847 (53)
80–84 years	330 (10)	848 (10)	246 (11)	378 (11)
85–89 years	169 (5)	348 (4)	101 (4)	151 (4)
≥ 90	51 (2)	67 (1)	48 (2)	45 (1)
Mean (±SD)	70 ± 10	70 ± 9	69 ± 10	70 ± 9
Prescriber of first 5-ARI				
Urologist	1,705 (54)	5,053 (58)	653 (29)	1,412 (40)
GP	1,063 (34)	2,242 (26)	1,275 (56)	1,546 (44)
Other	379 (12)	1,380 (16)	336 (15)	559 (16)
Prior use of urological care				
Yes	2,055 (65)	6,164 (71)	943 (42)	1,946 (55)
History of bladder or kidney stones				
Yes	50 (2)	148 (2)	16 (1)	48 (1)
Comorbidities				
Hypertension	1,653 (53)	4,444 (51)	1,116 (49)	1,771 (50)
Hypercholesterolemia	1,060 (34)	2,948 (34)	662 (29)	1,094 (31)
Diabetes type I	42 (1)	113 (1)	50 (2)	73 (2)
Diabetes type II	322 (10)	930 (11)	209 (9)	372 (11)
Parkinson’s disease	42 (1)	148 (2)	41 (2)	50 (1)
Multiple sclerosis	1 (<0.5)	1 (<0.5)	0 (0)	2 (<0.5)
Chronic disease score				
0–3	1,402 (45)	3,987 (46)	1,137 (50)	1,634 (46)
4–7	939 (30)	2,516 (29)	619 (27)	1,017 (29)
≥ 8	806 (26)	2,172 (25)	508 (22)	866 (25)
Mean (±SD)	5 ± 4	5 ± 4	4 ± 4	5 ± 4
Adherence (MPR 5-ARI) (%)				
Mean (±SD)	84 ± 13	85 ± 13	81 ± 14	84 ± 13
Database follow-up after cohort entry date				
≥ 1 year	2,264 (100)	3,517 (100)	3,147 (100)	8,675 (100)
≥ 2 years	1,906 (84)	2,975 (85)	2,361 (75)	6,395 (74)
≥ 3 years	1,596 (70)	2,463 (70)	1,700 (54)	4,526 (52)
≥ 4 years	1,291 (57)	1,991 (57)	1,115 (35)	2,972 (34)
≥ 5 years	970 (43)	1,490 (42)	618 (20)	1,715 (20)

*SD* standard deviation, *IQR* interquartile range, *MPR* medication possession rate

### Incidence of outcomes

The incidence of BPH-related prostate surgery among men using finasteride ranged from 12 per 1,000 person-years among those using monotherapy prescribed by a GP to 472 per 1,000 person-years among those using combination therapy prescribed by a urologist (Table 2). For men using dutasteride this was 10 per 1,000 person-years among those using monotherapy prescribed by a GP to 248 per 1,000 person-years among those using combination therapy prescribed by a urologist. A transurethral resection of the prostate (TURP) was most common in all cohorts (data not shown).

**Table 2:** Hazard ratios of BPH-related prostate surgery among men with BPH using finasteride or dutasteride

	Finasteride			Dutasteride			Dutasteride vs finasteride	
	Men with BPH-related surgery	PY at risk	Incidence per 1,000 PY (95 % CI)	Men with BPH-related surgery	PY at risk	Incidence per 1,000 PY (95 % CI)	Hazard ratio	
	n (%)^f^			n (%)^f^			Crude (95 % CI)^a^	Adjusted (95 % CI)
Overall							0.83 (0.62–1.10)	0.75 (0.56–0.99)^b^
Monotherapy	86 (4)	3,132	28 (22–34)	108 (3)	4,356	25 (20–30)	0.85 (0.64–1.13)	0.73 (0.54–0.98)^c^
Combination therapy	317 (9)	5,646	56 (50–63)	767 (9)	12,685	61 (56–65)	0.91 (0.80–1.04)	0.85 (0.74–0.97)^c^
Prescriber: GP								
Monotherapy	14 (1)	1,201	12 (6–20)	9 (1)	915	10 (5–19)	0.91 (0.39–2.13)	-^g^
Combination therapy	44 (4)	1,710	26 (19–35)	74 (4)	2389	31 (24–39)	1.07 (0.74–1.56)	1.10 (0.76–1.60)^d^
Prescriber: Urologist								
Monotherapy	26 (11)	142	183 (120–268)	45 (7)	433	104 (76–139)	0.56 (0.35–0.92)	0.77 (0.46–1.30)^d^
Combination therapy	105 (24)	223	472 (386–571)	272 (16)	1097	248 (219–279)	0.53 (0.42–0.66)	0.62 (0.50–0.78)^e^

*PY* person-years, *CI* confidence interval, *GP* general practitioner; ^a^Adjusted for geographic location; ^b^Adjusted for geographic location, cohort (mono- or combination therapy), adherence with 5-ARI treatment, prescriber, chronic disease score and number of GP visits); ^c^Adjusted for geographic location, adherence with 5-ARI treatment, prescriber and number of GP visits; ^d^Adjusted for geographic location and adherence with 5-ARI treatment; ^e^Adjusted for geographic location, adherence with 5-ARI treatment and number of drug dispensings; ^f^percentage of patients with an event in the specific group; ^g^None of the covariates were associated with BPH-related prostate surgery or 5-ARI treatment

**Fig. 1:**
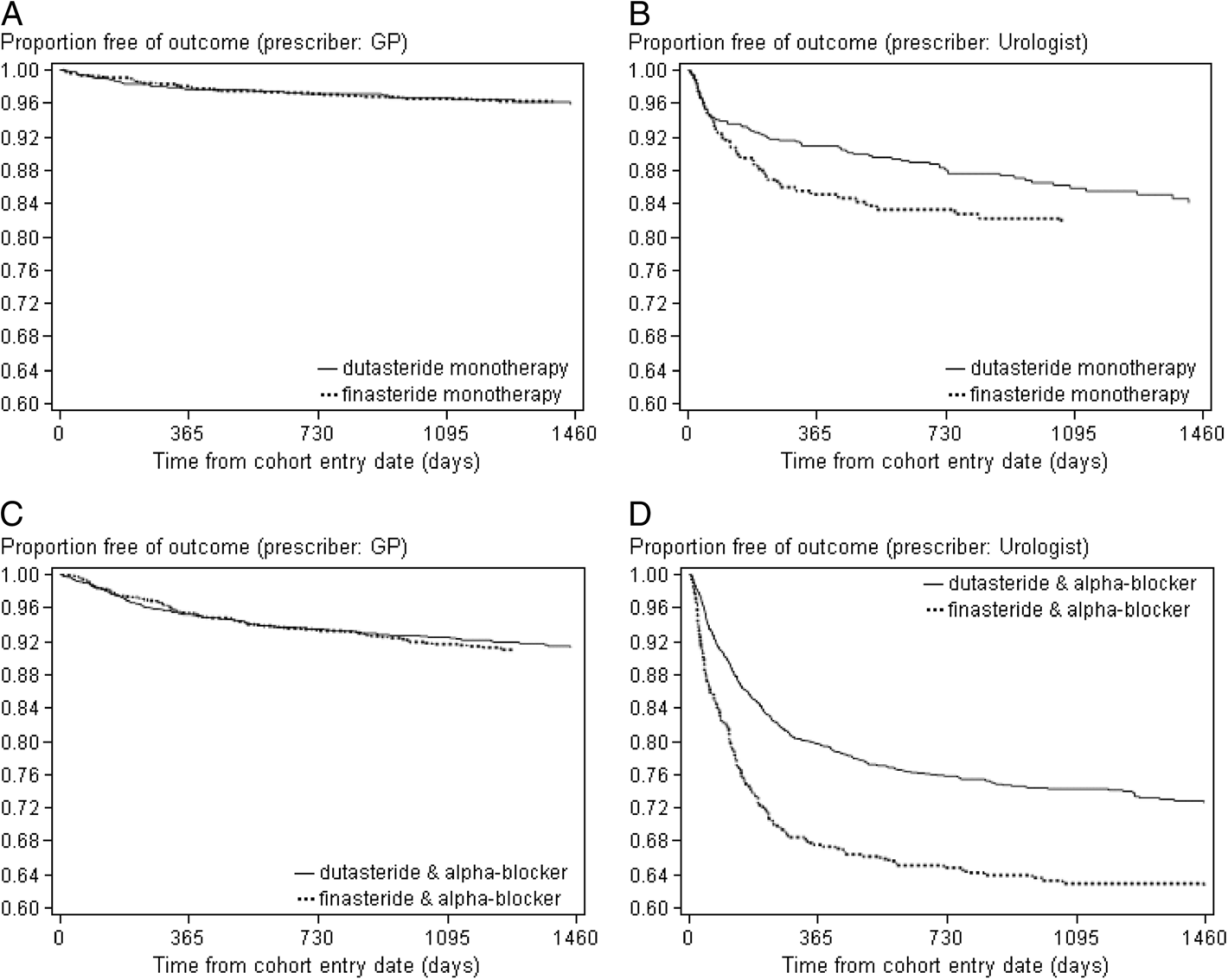
Kaplan-Meier survival curve showing the proportion of men free of BPH-related prostate surgery, stratified by type of initial BPH treatment and prescriber. **a**) dutasteride or finasteride monotherapy prescribed by GP, **b**) dutasteride or finasteride monotherapy prescribed by urologist, **c**) dutasteride or finasteride & alpha-blocker prescribed by GP, **d**) dutasteride or finasteride & alpha-blocker prescribed by urologist

Figure 1 shows the proportion of men free of BPH-related prostate surgery over time, stratified by type of initial BPH treatment and prescriber. Overall, dutasteride users had a lower risk of BPH-related prostate surgery than finasteride users. The crude HR was 0.83 (95 % CI: 0.62–1.10), however when adjusted for geographic location, cohort (mono- or combination therapy), adherence with 5-ARI treatment, prescriber, chronic disease score and number of GP visits the HR was 0.75 (95 % CI: 0.56–0.99). The strongest confounder was the initial prescriber. This lower risk was seen for men on monotherapy (adjusted HR: 0.73; 95 % CI: 0.54–0.98) as well as combination therapy (adjusted HR: 0.85; 95 % CI: 0.74–0.97) (Table 2). This association, however, was only present among men with a first dispensing from a urologist (HR: 0.77; 95 % CI: 0.46–1.30 for men on monotherapy and HR: 0.62; 95 % CI: 0.50–0.78 for combination therapy), while there was no difference in the risk of BPH-related prostate surgery among men with a first dispensing from a GP. In a sensitivity analysis, BPH-related prostate surgery was determined during total BPH treatment (censoring upon changes in alpha-blocker use) and in another sensitivity analysis during 5-ARI treatment (regardless of alpha-blocker use) with a wash-out period of 6 months after discontinuation. The difference in incidence of BPH-related prostate surgery between mono- and combination therapy remained, but the association with type of 5-ARI was less clear.

Only 1 % of the finasteride or dutasteride users were admitted for AUR during the first 5-ARI treatment episode. The incidence rates were 6 per 1,000 person-years for men on dutasteride monotherapy, 5 per 1,000 person-years for men on finasteride monotherapy, 9 per 1,000 person-years for men on dutasteride combination therapy and 5 per 1,000 person-years for men on finasteride combination therapy. Due to the low number of men admitted for AUR, no HR could be calculated.

## Discussion

To our knowledge, this is the first population-based study to report the incidence of BPH-related prostate surgery and AUR among men using dutasteride or finasteride in the Netherlands. Disease severity was accounted for by stratifying the results by type of therapy (mono- or combination with alpha-blocker) and prescriber (GP or urologist) in the absence of information on symptom severity, flow rate parameter, serum PSA or prostate size. In line with previous retrospective studies it was confirmed that the risk of BPH-related prostate surgery was lower among men using dutasteride compared to men using finasteride. The association was observed with either mono- or alpha-blocker combination therapy, however only among men who received their prescription from a urologist. Both dutasteride and finasteride blocks the type 2 5-alpha reductase enzymes and converts testosterone into DHT, which makes them both effective treatments for BPH [6, 7]. Dutasteride also blocks the type 1 5-alpha reductase enzymes which may suggest the possibility of improved outcomes with this treatment. Furthermore, dutasteride has a longer half-life compared to finasteride (approximately 4 weeks vs 6 h), which also might lead to better efficacy with dutasteride [6, 20].

The incidence of BPH-related prostate surgery was higher among men on combination therapy, which may reflect the treatment choices based on perceived severity of the condition. Furthermore, the incidence of BPH-related prostate surgery was higher in men treated by urologists compared to those treated by GPs. This difference may be explained by the fact that the choice for surgery takes place in secondary care. According to Dutch guidelines, watchful waiting is advised for patients without complications and with mild symptoms and/complaints [21]. This is mainly based on the finding that the effectiveness of both drug and invasive treatment in these patients is limited, especially if it is weighed against the potential side effects or complications. Patients with severe symptoms who do not benefit enough from lifestyle changes should be treated with an alpha-blocker according to Dutch guidelines. When results of alpha-blocker treatment are unsatisfactory after 6 weeks, the medication should be stopped and the patients should be referred to a specialist to discuss the possibilities of invasive procedures. Treatment with a 5-alpha reductase inhibitor (finasteride or dutasteride) is an appropriate option for patients with moderate to severe complaints and a prostate volume >30–40 ml. If a fast reduction of complaints is desired, a 5-alpha reductase inhibitor can be given (temporarily) in combination with an alpha-blocker. When other possible causes of BPH are suspected, for example prostate cancer, the primary care guidelines refer the patient to a specialist for additional examination [21]. Furthermore, when the symptoms are severe and surgical treatment is the only option, the patient will be directly referred to a specialist. Urologists have the choice to recommend surgical treatment and conduct the surgery themselves. This is not the case for GPs, who have to refer patients when a BPH-related prostate surgery is needed. As a result, BPH patients in primary care are less severe and patients have less room for improvement. This was also shown in a study performed among four GP practices in the Netherlands. In this study, it was shown that 30 % of the BPH patients were immediately referred to the urologist. For the majority of the patients, the main reason for referral was the nature and the severity of the micturition, a suspicious rectal toucher or requested by the patient [22].

Until the early 90s, the choice of therapy in patients with BPH consisted of either watchful waiting or surgical treatment. In the last 15 years, many new therapies were introduced of which alpha-blockers and 5-ARI are most common.

Extending the follow-up with 6 months after discontinuation of 5-ARI affected the incidence rates only marginally. Applying a more strict definition of treatment, i.e. not only continuous 5-ARI treatment but also no change in alpha-blocker therapy, resulted in a more pronounced difference in rates between mono- and combination therapy, but the difference between dutasteride and finasteride users did not change.

Finasteride became available on the European market in 1992, while dutasteride became available in 2002. As new drugs are usually first given to a selected group of patients, mostly the more severe, this may create a bias in favor of older products and thus a confounded comparison of the risks of BPH-related prostate surgery between dutasteride and finasteride. In order to assess this potential introduction bias, a sensitivity analysis was performed excluding men starting with 5-ARI between 2003 and 2006. However, excluding those years did not change the comparison of risk of BPH-related prostate surgery between dutasteride and finasteride. Therefore, introduction bias seems unlikely. Furthermore, for this study, data up to and including 2011 was used. As in more recent years no substantial changes occurred with respect to the management and treatment of BPH, major changes in the association between type of 5-ARI and BPH-related prostate surgery are not likely.

Consistent with the current results, a previous study showed that dutasteride was associated with a lower risk of BPH-related surgery (HR 0.75; 95 % CI: 0.58–0.98) [13]. However, whether the patients in this study were on monotherapy or combination therapy was not specified. In another study among men aged ≥50 years, no significant difference in surgery rates was found between patients using dutasteride or finasteride [12].

AUR was a relatively infrequent event in this study, occurring in only 1 % of the men using finasteride or dutasteride. AUR was probably underreported in this study because the database did not capture emergency department visits. In a previous study among patients aged 50 years or older diagnosed with BPH, the rate of AUR after 5 to 12 months was significantly lower in the dutasteride group compared with the finasteride group (5.3 % vs 8.3 %) [12].

## Conclusions

In conclusion, the risk of BPH-related prostate surgery was lower among men using dutasteride than among men using finasteride. This association was present among men treated with 5-ARI monotherapy as well as men treated with 5-ARI combination therapy. The difference in surgery rates was limited to BPH patients treated in secondary care.

## Abbreviations

5-ARIs: 5-alpha reductase inhibitors
ATC: Anatomical therapeutic chemical
AUR: Acute urinary retention
BPH: Benign prostatic hyperplasia
CDS: Chronic disease score
CI: Confidence interval
DHT: Dihydrotestosterone
GP: General practitioner
HR: Hazard ratio
ICPC: International Classification of Primary Care
IPSS: International Prostate Symptom Score
IQR: Interquartile range
LUTS: Lower urinary tract symptoms
MPR: Medication possession rate
PSA: Prostate-specific antigen
PY: Person-years
SD: Standard deviation
WHO: World Health Organization
